# Osteopontin, WNT3A, and ABCB5 Biomarkers Expression in Osteosarcoma Patients

**DOI:** 10.1055/s-0044-1788671

**Published:** 2024-09-04

**Authors:** Glauco José Pauka Mello, Cleima Coltri Bittelbrunn, Glauco Vinicius Bittelbrunn Pauka Mello, Fernanda Pinto Garcia, Ana Valéria Brunetti Rigolino, Pedro Reggiani Anzuatégui

**Affiliations:** 1 Serviço de Ortopedia e Traumatologia, Hospital Erasto Gaertner, Curitiba, PR, Brasil; 2 Centro de Ortopedia e Fisioterapia Batel, Curitiba, PR, Brasil; 3 Pontifícia Universidade Católica do Paraná, Curitiba, PR, Brasil

**Keywords:** biomarkers tumor, immunohistochemistry, osteopontin, osteossarcoma

## Abstract

**Objective** This study aimed to correlate the expression, by immunohistochemistry, of the proteins OPN, ABCB5, and WNT3A from anatomopathological materials obtained from paraffin blocks, slides, or both, from patients with osteosarcoma (OS), analyzing epidemiological characteristics, as well as their presence and influence on the evolution and progression of the disease.

**Methods** After the initial case selection, we searched for the respective paraffin blocks and took only those with sufficient tumor mass to allow additional sections with no complete loss of biological material. The sarcoma area identification in representative paraffin blocks used multisample blocks (tissue microarray [TMA]) created on a BenchMark ULTRA (Roche Diagnostics Corporation, Indianapolis, IN, USA) instrument. Then, we analyzed the association between the expression of ABCB5, WNT3A, and osteopontin (OPN) markers with the variables age, location, and tissue type (Fisher exact test/Chi-squared test).

**Results** The average age of the patients was 23 years, and the rate of males and females was the same. We analyzed 40 slides from 28 OS patients seen from 2005 to 2017. Their follow-up time was 80.0 months, and the 5-year survival rate was 46.7%. Most metastases occurred in lung tissue (92.9%). Proteins ABCB5, OPN, and WNT3A did not present statistical significance when compared with age group, neo-adjuvant, adjuvant, or both, chemotherapy, location, survival, or death. Osteopontin was negative in all samples. WNT3A expression occurred in patients who died early.

**Conclusion** In an immunohistochemical study, ABCB5, OPN, and WNT3A did not have statistical significance. In the parameters analyzed, they did not seem to be a predictive or aggressive factor for OS.

## Introduction

This article addresses the study of osteopontin (OPN), WNT3A, and ABCB5 as biomarkers in osteosarcoma (OS) patients.

When analyzed in other tumors, especially carcinomas, the ABCB5 biomarker has a critical role on the evaluation of resistance to chemotherapy agents commonly used in OS treatment.

The WNT3-signaling pathway is common in several tumors, including carcinomas and sarcomas. This pathway plays a significant role in intracellular process regulation, and its presence in different tumor types highlights its importance in several contexts.

Although OPN is present in many cases of carcinoma, its crucial function in bone tissue relates to the preosteoblast maturation into osteoblast. This transformation is key for osteosarcoma presentation, emphasizing the complex role of osteopontin, especially in bones.

There are no known biomarkers capable of revealing the presence of bone tissue sarcomas, and their aggressiveness and propensity to metastasize. The opportunity to explore and study the biomarkers analyzed in the present article has presented itself, and we are taking this chance to integrate our experience into this investigation. This is a crucial step towards deepening our understanding of the nature of these sarcomas, significantly contributing to advancing the diagnosis and treatment of these complex conditions.

In OS, a highly aggressive form of bone sarcoma, biomarkers play distinct and crucial roles in tumor progression. The ABCB5 gene, recognized as a biomarker associated with therapeutic resistance, modulates the activity of the WNT3-signaling pathway, contributing to supporting the proliferative capacity and cellular resistance in OS. The WNT3-pathway, an intrinsic biomarker, regulates the expression of genes related to uncontrolled bone growth and carcinogenesis.

Simultaneously, OPN, also considered a biomarker, suffers influences from other factors, including the WNT3 pathway, and it is implicated in bone formation, playing a significant role in bone tumorigenesis. The expression and activity of biomarkers such as ABCB5, the WNT3-signaling pathway, and OPN are associated with OS aggressiveness.

Studies observed that high levels of these biomarkers often correlate with more aggressive disease behavior. The presence of ABCB5, for instance, has been associated with resistance to conventional treatments, potentially contributing to OS progression. Abnormal activation of the WNT3 pathway is related to uncontrolled tumor growth and invasiveness, while increased OPN expression has been associated with a greater capacity for metastasis.

Therefore, analyzing these biomarkers can offer prognostic insights, since they are widely studied in lung and breast carcinomas and melanomas.

Identifying and understanding these biomarkers are essential to guide more precise and effective therapeutic strategies against OS. The biggest difficulty is finding studies with a significant number of cases.

Approximately 8.7 per million children and adolescents under the age of 20 suffer from OS, which originates in the mesenchymal tissue. As such, this is the most common malignant tumor in the age group up to 20 years.

The definitive diagnosis relies on the clinical presentation of pain and swelling, followed by radiological imaging investigation, biopsy confirmation, or both.

The survival rates, in a 5-year time range, are higher in patients with no metastases. This depends on the biological behavior of the condition.

Most metastases occur in lung tissues. The advent of neoadjuvant polychemotherapy followed by surgical treatment and adjuvant chemotherapy improved survival by around 50 to 70% depending on the heterogeneous nature of this disease.

Several prognostic factors with biomarkers have already been researched for OS. However, studies found no biomarker with strong evidence for diagnosing or evaluating prognoses, especially from metastatic diseases, and identifying potential therapeutic targets. Our study highlights WNT3A, OPN, and ABCB5 as biomarkers.

Laboratory experiments revealed that WTN3A is a protein from the canonical WNT family, the most involved in abnormal ß-catenin levels in the cell nucleus. 1 In normal bone tissue, OPN is vital for osteoblast development. Furthermore, ABCB5 is a novel human membrane transporter from the ABC protein group, identified and characterized in human skin.

This study aimed to correlate the expression, by immunohistochemistry, of OPN, ABCB5, and WNT3A from anatomopathological materials obtained from OS patients' paraffin blocks and/or slides, analyzing epidemiological characteristics, their presence, and influence on the evolution and progression of the disease.

## Materials and Methods

The institutional ethics committee approved this study under number CAAE 69385417.7.1001.0103.

We obtained clinical and anatomical data from the electronic medical records of the hospital's Philips Tasy Electronic Medical Record (Philips Healthcare, Best, Netherlands) system and reviewed the physical records and pathological anatomical reports from the pathological anatomy service from the 64 patients with OS treated from 2005 to 2017.

The initial search used the International Classification of Diseases (ICD) code C40 and collected the clinical notes from each case in a standardized protocol with the following variables: name, patient age at diagnosis, gender, number of tumors, paraffin block number, histological diagnosis, primary tumor location, date of diagnosis, presence of metastases, metastasis location at diagnosis (if any), survival time in months, death (if any), follow-up time, and disease progression.

The end date for assessing patient survival was December 2017.

### Disease Progression and Survival Time

Some patients, even during treatment, experienced tumor recurrence or disease progression with clinical staging worsening, either due to the tumor recurrence at the surgical site or the appearance of local (skip) or distant metastasis in the viscera or lymph nodes. Distant metastasis occurred in the lungs and lymph nodes.

Subsequently, we calculated the disease-free time in months, that is, the period during which the patient remained with no signs or symptoms of the neoplasm.

We calculated the survival time by subtracting the date of OS diagnosis from the date of death or last assessment. The date in which the last OS diagnosis was given was subtracted from the date of death or last follow-up exam.

#### Sample Selection

After the initial selection of cases, we searched for the respective paraffin blocks and took only those with sufficient tumor mass to allow additional sections with no complete biological material loss.

A second independent pathologist reevaluated the tumor slides to confirm OS diagnosis.

We requested hematoxylin and eosin (HE)-stained slides from blocks, in case of their unavailability. If more than one paraffin block was available, the pathologist chose the one with the larger neoplastic mass.

We sent the duly-checked blocks to OPN, WNT3A, and ABCB5 biomarker immunostaining.

#### Immunohistochemistry

The immunohistochemistry technique used the Ventana BenchMark ULTRA (Roche Diagnostics Corporation, Indianapolis, IN, USA) instrument with integrated 3-in-1 processing. Preparation occurred in the following order: deparaffinization; rehydration; antigen retrieval with the Cell Conditioning 1 (high pH) and 2 solutions (Roche Diagnostics Corporation) (low pH) buffers; primary antibody incubation for 16 to 20 minutes at room temperature; immunoperoxidase technique; staining amplification using the UltraView Universal DAB Detection Kit (Ventana Medical Systems, Oro Valley, AZ, USA). After immunostaining, we analyzed the tissue microarray (TMA) slides. Internal and external positive controls tested the fidelity of the reactions.

### Antibodies Employed

Table 1 describes the primary antibodies employed in this study.

**Table 1:** Description of primary antibodies, their respective manufacturers, and dilutions

Biomarker	Primary antibody	Manufacturer	Dilution
ABCB5	Anti-ABCB5, clone 5H3C6	*GeneTex*	1:200
OPN	Anti-Osteopontin, policlonal	*Anti-Osteopontin, policlonal*	1:25
WNT3A	Anti-WNT3A, policlonal	*GeneTex*	1:400

**Abbreviation:** OPN, osteopontin. **Source:** The authors (2019).

### Immunostaining Report

Two different pathologists reviewed the slides at distinct times using an Olympus CX31 (Olympus Life Science, Waltham, MA, USA) microscope and classified them per the following parameters:

- **Positive:** Presence of anti-ABCB5 antibody in the cytoplasmic membrane and the cytoplasm; presence of anti-WNT3A antibody in the cytoplasm; c-staining; and presence of anti-OPN antibody in the cytoplasm.
- **Negative:** Lack of chromogenic staining expression for antibody targets.
- **Inconclusive:** Impossibility of evaluating antibody expression due to pre-analytical problems (poor fixation, acid decalcification, and inadequate paraffin).

## Results

Information regarding the patients included medical record data, patient number, slides, age at diagnosis, gender, and tumor location per the sample studied ( Table 2 ). We analyzed 40 samples from paraffin blocks of the primary and metastatic tumors' biopsy and surgical specimens, taken from a total of 28 OS patients. These samples were from the anatomical pathology service and dated from 2005 to 2017. We correlated survival, presence or absence, metastasis site, and expression of ABCB5, WNT3A, and OPN markers ( Table 3 ).

**Table 2:** Data: medical record, patient number, slides, age at diagnosis, gender, and location

Medical record	Patient number	Slides	Age at diagnosis	Gender	Location
14001945	4	162818B	20	Male	Inferior limb
	4	167536B / 2-17	20	Male	Inferior limb
14002913	5	143741B	12	Female	Superior limb
1001989	6	143042B	26	Female	Inferior limb
	6	149162B / 3-11	26	Female	Inferior limb
	6	157099B	27	Female	Inferior limb
13000565	7	130884B	13	Female	Inferior limb
	7	135117	13	Female	Inferior limb
	7	140955B / 1-5	14	Female	Inferior limb
	7	143457B	14	Female	Axial skeleton
12006205	8	140430B	62	Male	Superior limb
13005621	10	138466B 1-57	19	Male	Inferior limb
13002093	11	134093B	20	Male	Inferior limb
	11	135736B	20	Male	Inferior limb
7002221	12	073238B / 1-2	16	Female	Superior limb
	12	073801B	16	Female	Superior limb
7002774	13	074648B	16	Male	Inferior limb
	13	077531B	16	Male	Inferior limb
6008700	14	0608869E	61	Female	Inferior limb
7001977	15	074428B	36	Female	Inferior limb
11003053	34	116772B / 1-8	20	Male	Inferior limb
11004802	36	116711B	45	Male	Superior limb
13001519	38	135295B / 1-25	13	Female	Inferior limb
5006762	40	056664B	13	Female	Inferior limb
6002708	43	0602903B	10	Female	Inferior limb
6001225	45	0606133B / 1	13	Female	Inferior limb
6008407	47	070309B / 1-3	27	Male	Inferior limb
5004056	48	0600157B / 1-2	23	Male	Superior limb
15000164	51	150432B / 1-2	21	Female	Inferior limb
14006899	52	154918B / 2-7	18	Male	Inferior limb
14007558	53	150955B / 3-20	14	Female	Inferior limb
15006646	55	167192B / 1-15	26	Female	Axial skeleton
15007598	56	1610793B / 2-6	32	Female	Inferior limb
16002529	58	163780B	20	Male	Superior limb
	58	168657B / 1-14	20	Male	Superior limb
16004018	59	166003B / 1-9	22	Male	Superior limb
16009072	62	1612817B / 1-2	16	Male	Inferior limb
	62	170557B / 1-13	17	Male	Inferior limb
17008495	63	1714419B	11	Male	Inferior limb

**Table 3:** Data: survival, metastasis, location, ABCB5, WNT3A, and OPN

Medical record	Survival (months)	Metastasis	Location	ABCB5	WNT3A	OPN
14001945	57	Yes	Lung	Inconclusive	Inconclusive	Inconclusive
	52	Yes	Lung	Positive	Negative	Negative
14002913	67	No		Negative	Negative	Negative
1001989	18	Yes	Lung	Positive	Negative	Negative
	12	Yes	Lung	Negative	Negative	Negative
	2	Yes	Lung	Negative	Negative	Negative
13000565	17	Yes	Lung	Positive	Negative	Negative
	12	Yes	Lung	Positive	Negative	Negative
	5	Yes	Lung	Positive	Positive	Negative
	3	Yes	Lung	Inconclusive	Inconclusive	Inconclusive
12006205	72	No		Positive	Negative	Negative
13005621	3	No		Inconclusive	Inconclusive	Inconclusive
13002093	11	Yes	Lung	Positive	Positive	Negative
	12	Yes	Lung	Negative	Negative	Negative
7002221	24	Yes	Lung	Inconclusive	Inconclusive	Inconclusive
	24	Yes	Lung	Positive	Negative	Negative
7002774	29	Yes	Lung	Positive	Positive	Negative
	25	Yes	Lung	Positive	Negative	Negative
6008700	16	No		Negative	Negative	Negative
7001977	124	No		Negative	Negative	Negative
11003053	75	Yes	Lung	Positive	Negative	Negative
11004802	68	No		Positive	Negative	Negative
13001519	8	Yes	Lung	Positive	Negative	Negative
5006762	41	Yes	Lung	Inconclusive	Inconclusive	Inconclusive
6002708	128	No		Positive	Negative	Negative
6001225	16	Yes	Lymph node	Positive	Negative	Negative
6008407	118	Yes	Lung	Inconclusive	Inconclusive	Inconclusive
5004056	133	No		Positive	Negative	Negative
15000164	55	Yes	Lung	Negative	Negative	Negative
14006899	3	No		Negative	Negative	Negative
14007558	34	Yes	Lung	Negative	Negative	Negative
15006646	2	No		Inconclusive	Inconclusive	Inconclusive
15007598	52	No		Positive	Negative	Negative
16002529	18	Yes	Lung	Inconclusive	Inconclusive	Inconclusive
	13	Yes	Lung	Positive	Negative	Negative
16004018	36	No		Inconclusive	Inconclusive	Inconclusive
16009072	12	No		Positive	Negative	Negative
	11	No		Negative	Inconclusive	Inconclusive
17008495	24	No		Negative	Negative	Negative

**Abbreviation:** OPN, osteopontin.

Table 4 presents the analysis performed based on data from 40 slides of 28 OS patients.

**Table 4:** Pre and postchemotherapy slides

Patient	Total	Number of slides	
		Treatment-free bone tissue	Posttreatment bone tissue
1	1	1	
2	1	1	
3	1		1
4	1	1	
5	1	1	
6	1	1	
7	1	1	
8	1		1
9	1	1	
10	1	1	
11	1		1
12	1		1
13	1		1
14	1	1	
15	1		1
16	1		1
17	1		1
18	1		1
19	1	1	
20	2	1	1
21	2	2	
22	2	1	1
23	2	2	
24	2	1	1
25	2	1	1
26	2	2	
27	3	1	2
28	4	1	3
Total slides	40	22	18

Table 5 shows patient-related variables, such as age at diagnosis, gender, number of slides throughout the follow-up period, and deaths.

**Table 5:** Patient-related variables ( *n* = 28)

Variable	Valid n	Classification	Result*
Age at diagnosis (years)	28		23 ± 13.4 (10–62)
		Up to 20	17 (60.7)
		Over 20	11 (39.3)
Gender	28	Female	14 (50)
		Male	14 (50)
Number of slides during follow-up period	28	1	19 (67.9)
		2, 3, or 4	9 (32.1)
Death	28	No	14 (50)
		Yes	14 (50)
Follow-up time (no death) (months)	14		80.0 ± 42.0 (27.5–150)
Survival time (death) (months)	14		18.2 ± 12.1 (2.7–42.1)
General follow-up time (months)	28		48.9 ± 43.6 (2.7–150)
Metastasis	28	No	14 (50)
		Yes	14 (50)
Metastasis location (per patient)	14	Lung	13 (92.9)
		Lymph node	1 (7.1)

**Note:** *Mean ± standard deviation (minimum–maximum) or frequency (percentage).

Table 6 reports the survival percentages at each time point per the Kaplan-Meier estimative.

**Table 6:** Survival rates at up to 5 years

Time (months)	Survival (%)
0 (diagnosis)	100%
3 months	100%
6 months	96.4%
1 year	92.9%
1.5 years	67.9%
2 years	64.3%
3 years	56.9%
4 years	52.5%
5 years	46.7%

### Descriptive Data from ABCB5, WNT3A, and OPN Biomarkers

The results (valid n) included tumor location (lower limbs, upper limbs, axial skeleton), slide type (biopsy, postchemotherapy, or with no chemotherapy), and slide tissue type (treatment-free and after therapy). We considered a single slide per patient, obtaining the following results (n = 28): tumor location – lower limbs (n [%], 20 [71.4]), upper limbs (n [%], 7 [25.0]), axial skeleton (n [%], 1 [3.6]); slide type – biopsy (n [%], 16 [57.1]), postchemotherapy (n [%], 10 [35.7]), no chemotherapy (n [%], 2 [7.1]); tissue type at the slide – treatment-free bone tissue (n [%], 18 [64.3]), posttreatment bone tissue (n [%], 10 [35.7]).

Descriptive data regarding ABCB5, WNT3A, and OPN considering a single slide per patient and percentages and after removing inconclusive results, are the following:

#### ABCB5, valid n = 28

Negative (n [%], 7 [25.0])

Positive (n [%], 14 [50.0])

Inconclusive (n [%], 7 [25.0])

#### ABCB5, valid n = 21

Negative (n [%], 7 [33.3])

Positive (n [%], 14 [66.7])

#### WNT3A, valid n = 28

Negative (n [%], 19 [67.9])

Positive (n [%], 2 [7.1])

Inconclusive (n [%], 7 [25.0])

#### WNT3A, valid n = 21

Negative (n [%], 19 [90.5])

Positive (n [%], 2 [9.5])

#### OPN, valid n = 28

Negative (n [%], 21 [75.0])

Inconclusive (n [%], 7 [25.0])

#### Assessment of Factors Associated with Biomarker Results and Between Factors and Survival

For this analysis, the observation unit was the slide. Slides from the same patient were considered independent units.

For each variable, tissue type, tumor location, and biomarker analyzed, we tested the null hypothesis that there is a lack of association between the variable and the marker versus the alternative hypothesis, that is, the presence of an association.

For age at diagnosis and each marker, we tested the null hypothesis that the mean age is the same for all markers versus the alternative hypothesis, that is, that the mean age is different.

Regarding survival, we tested the null hypothesis which was lack of association between each variable and survival, versus the alternative hypothesis, that is, the presence of an association. These are percentages of deaths according to the variable age at diagnosis, gender, metastases, tumor site, ABCB5, WNT3A, and OPN markers, and *p* -values from statistical tests.

## Discussion

The selected OS patients were classified as Enneking grade IIB or III.

In the analysis of the epidemiological profile, 17 patients were under 20-years-old (young patients), and 11 were over 20-years-old. The youngest subject was 10-years-old, which is consistent with the literature. 2 3 4 5 In this sample, the male and female incidence was the same, at 50%. Studies with larger samples showed a slight male predominance. 6

Among the study's patients, regarding metastasis location: 14 patients with metastasis, 13 (92.9%) of whom had metastases in the lung tissue and 1 (7.1%) in the lymph nodes. 2 In this study, 50% of the subjects developed lung metastases. According to Evola et al., 7 approximately 20% of patients had lung metastases at the initial diagnosis, 40% had metastases at a later stage, and 80% of metastases occurred in the lung.

The 5-year (60-month) survival rate was 46.7%, slightly below literature reports, which range from around 50 to 65%. 2 3 8 Death occurs between the first and third years postdiagnosis in most patients, indicating that it is a very aggressive condition. 5

The tumor was in the lower limbs in 20 patients (71.4%), in the upper limbs in 7 (25%), and in the axial skeleton in 1 (3.6%). These rates are consistent with the literature. 2 5 7 9 10

The epidemiological variables (age at diagnosis, gender, metastases, location, and biomarkers) had no statistical significance when analyzing death as an outcome. The metastasis variable trends towards significance ( *p* = 0.086); however, the sample is too small for this conclusion.

The ABCB5 marker had positive expression in 14 patients (66.7) and negative expression in 7 subjects (33.3%) after excluding the 7 inconclusive results. The biomarker WNT3A had positive expression in 2 patients (9.5%) and negative expression in 19 (90.5%), after removing the inconclusive ones. The OPN marker was always negative, with no expression either in slides of tissues receiving neoadjuvant chemotherapy or not.

When evaluating the expression of markers ABCB5 and WNT3A versus OS location in the upper and lower limbs and age groups below or above 20 years, positive or negative staining had no significance. However, the OPN marker was negative in the upper and lower limb slides and in the age groups above and below 20 years.

When evaluating the slides from the 28 eligible patients, 6 had slides from pre and postchemotherapy samples. The pretreatment evaluation had one slide per patient. For postchemotherapy evaluations, slides were analyzed according to disease progression events (relapses and metastases), which resulted in the analysis of new slides referring to these events. Therefore, the number of posttreatment slides (9) was higher than that of pretreatment slides.

For the ABCB5 marker in the pretreatment evaluation, 3 slides (50%) were positive, none was negative (0%), and 3 were inconclusive (50%). After chemotherapy treatment, 6 were positive (66.7%), 2 were negative (22.2%), and only 1 was inconclusive (11.1%). This biomarker assesses resistance to chemotherapy drugs, being a chemoresistance mediator, identified and proven to be critical and specific to the drug doxorubicin. In the present sample, patients under chemotherapy regimens also received doxorubicin. Therefore, this protein expression was detectable in 50% of pretreatment slides and 66.7% of posttreatment slides. This finding highlights the hypothesis that tumor stem cells develop the capacity to resist cytotoxic drugs, hindering the inhibition of tumor progression. 11 12 13

Haydon et al. 14 concluded that deregulation of the WNT/ß-catenin pathway is very common in OS. However, in this sample, only two patients showed signs of activation of this pathway. Nevertheless, when this occurred, it was precisely in patients with the worst prognosis. There was no statistical significance when considering the expression of this protein influencing survival.

There was no OPN detection in osteoblastomas and bone remodeling specimens. Its expression did not influence overall patient or disease-free survival. It does not provide predictive information about the outcome of OS patients. The formation and differentiation of osteoblasts are fundamental for bone tissue development. Furthermore, OPN interferes with the differentiation of the primary mesenchymal cell into preosteoblasts, generating mature osteoblasts. Disruption of this process may be the main cause of OS. Luo et al. 15 reported a decrease in OPN levels in OS, as it does not act in the osteoblast differentiation. This protein's expression was negative in 100% of the slides, which is consistent with the findings of these authors.

## Conclusion

In an immunohistochemical study of OS, the biomarkers ABCB5, OPN, and WNT3A showed no statistical significance regarding epidemiological parameters. They did not prove to be a predictive or aggressive factor for OS. A slight increase in ABCB5 was observed postchemotherapy. Blocking ABCB5-protein expression supports the hypothesis of increased survival free from disease recurrence. The OPN protein was present in the etiology and development of OS. In this study, the canonical WNT/ß-catenin pathway was not convincingly present in the expression of the WNT3A marker. Further prospective studies with frozen, randomized, and controlled tumor bank material are required to consolidate and promote advances in future research.
