# Supplementary material for: Osteopontin, WNT3A, and ABCB5 Biomarkers Expression in Osteosarcoma Patients
Source: Rev Bras Ortop (Sao Paulo). 2024 Sep 4;59(4):e599–606. doi: 10.1055/s-0044-1788671 (PMC11374396; doi:10.1055/s-0044-1788671)
Supplement: Related texts

# Research article: Expressão dos biomarcadores osteopontin, WNT3A e ABCB5 em pacientes com osteossarcoma

**Keywords:** biomarcadores tumorais, imuno-histoquímica, osteopontina, osteossarcoma

## Abstract

**Objetivo** Correlacionar a expressão, por imunoistoquímica, das proteínas OPN, ABCB5 e WNT3A de material anatomopatológico, obtido de blocos de parafina e/ou lâminas, em pacientes com osteossarcoma (OS), analisando as características epidemiológicas, sua presença e influência na evolução e progressão da doença.

**Métodos** Após a seleção inicial dos casos, ocorreu a busca dos respectivos blocos de parafina, dentre os quais foram selecionados somente aqueles que possuíam massa tumoral suficiente para serem realizados cortes adicionais sem que todo o material biológico fosse utilizado. Foram identificadas áreas do sarcoma nos blocos de parafina representativos para a confecção de blocos multiamostrais (microarranjo de tecidos, ou *tissue microarray* [TMA], em inglês), realizada em instrumento *BenchMark ULTRA* (Roche Diagnostics Corporation, Indianapolis, IN, USA). Foi então analisada a associação entre a expressão dos marcadores ABCB5, WNT3A e OPN com as variáveis idade, localização e tipo de tecido (teste exato de Fisher/Qui-quadrado).

**Resultados** A média de idade foi de 23 anos, e a incidência de pacientes dos sexos masculino e feminino foi a mesma; foram analisadas 40 lâminas de 28 pacientes com OS, entre 2005 e 2017, com tempo de segmento de 80,0 meses, e o tempo de sobrevida foi de 46,7% em 5 anos. Metástases ocorreram em tecido pulmonar (92,9%). Quanto aos marcadores ABCB5, OPN e WNT3A, não apresentaram significância estatísticas quando comparados com faixa etária, neo-adjuvância e/ou adjuvância quimioterápica, localização, sobrevida ou óbito. O OPN mostrou-se negativo em todas as amostras. E o WNT3A expressou-se nos pacientes com óbitos precoces.

**Conclusão** As proteínas ABCB5, OPN e WNT3A, em estudo imunoistoquímico, não se mostraram presentes com significância estatística. Nos parâmetros analisados, não surgem como sendo fatores preditivos ou de agressividade para o OS.

## Introdução

O presente trabalho aborda o estudo dos marcadores osteopontina (OPN), WNT3A, e ABCB5 em pacientes com osteossarcoma (OS).

O biomarcador ABCB5, ao ser analisado em outros tumores, especialmente carcinomas, desempenha um papel crucial na avaliação da resistência ao tratamento com agentes quimioterápicos comumente utilizados no combate ao OS.

A via de sinalização WNT3 é uma ocorrência comum em diversos tumores, abrangendo tanto carcinomas quanto sarcomas. Essa via desempenha um papel significativo na regulação de processos intracelulares, e sua presença em diferentes tipos de tumores destaca sua importância em contextos variados.

Quanto ao biomarcador OPN, embora esteja presente em muitos casos de carcinoma, sua função crucial no tecido ósseo está relacionada à maturação do pré-osteoblasto em osteoblasto. Essa transformação é um ponto crucial na apresentação do osteossarcoma, enfatizando a complexidade do papel desempenhado pela OPN, especialmente no contexto ósseo.

Não são conhecidos biomarcadores capazes de revelar a presença da doença, sua agressividade e propensão à formação de metástases. A oportunidade de explorar e estudar os biomarcadores analisados neste artigo se apresentou, e estamos aproveitando esta chance para integrar nossa experiência a essa investigação. Este é um passo crucial para aprofundar o entendimento da natureza desses sarcomas, visando contribuir significativamente para o avanço no diagnóstico e tratamento dessa doença complexa.

No cenário do OS, uma forma altamente agressiva de sarcoma ósseo, os biomarcadores desempenham papéis distintos e cruciais na progressão tumoral. O gene ABCB5, reconhecido como um biomarcador associado à resistência terapêutica, modula a atividade da via de sinalização WNT3, contribuindo para a sustentação da capacidade proliferativa e resistência celular no OS. A via WNT3, um biomarcador intrínseco, regula a expressão de genes relacionados ao crescimento descontrolado e à carcinogênese óssea.

Simultaneamente, a OPN, também considerada um biomarcador, é influenciada por fatores, incluindo a via WNT3, e está implicada na formação óssea, desempenhando um papel significativo na tumorigênese óssea. A expressão e atividade dos biomarcadores como ABCB5, a via de sinalização WNT3e a OPN têm sido associadas à agressividade do OS.

Em estudos, observou-se que níveis elevados desses biomarcadores muitas vezes estão correlacionados com um comportamento mais agressivo da doença. A presença de ABCB5, por exemplo, foi associada à resistência a tratamentos convencionais, o que pode contribuir para a progressão do OS. A ativação anômala da via WNT3está relacionada ao crescimento descontrolado e à invasividade tumoral, enquanto a expressão aumentada de OPN tem sido associada a uma maior capacidade de metastização.

Portanto, a análise desses biomarcadores pode oferecer *insights* prognósticos, lembrando que esses biomarcadores são amplamente estudados em carcinomas tais como: pulmão, mama e melanoma, entre outros.

A identificação e compreensão desses biomarcadores são essenciais para orientar estratégias terapêuticas mais precisas e eficazes contra o OS. A maior dificuldade é encontrar estudos com um número de casos significativo.

Aproximadamente 8,7 por milhão de crianças e adolescentes com menos de 20 anos sofrem desta patologia, que se origina no tecido mesenquimal. Isso torna esse o tumor maligno mais frequente na faixa etária até 20 anos.

O diagnóstico definitivo depende da apresentação clínica de dor e aumento de volume, seguida de uma investigação de imagem radiológica e/ou confirmação por biópsia.

Sabemos que a sobrevida, em um intervalo de 5 anos, é maior em pacientes sem metástases. Isso depende do comportamento biológico da patologia.

A maioria das metástases ocorrem no tecido pulmonar. Com o advento da poliquimioterapia neoadjuvante seguida de tratamento cirúrgico e quimioterapia adjuvante, a sobrevida melhorou em torno de 50 até 70%, dependendo da natureza heterogênea desta patologia.

Vários fatores prognósticos com biomarcadores já foram pesquisados para o OS. Porém, ainda não foi encontrado um biomarcador com forte evidência em diagnosticar ou avaliar prognósticos, principalmente em doenças metastáticas, bem como identificar possíveis alvos terapêuticos. Neste estudo, iremos evidenciar os biomarcadores WNT3A, OPN e ABCB5.

O biomarcador WTN3A, em experimentos de laboratório, é a proteína da família WNT canônica que mais se mostra envolvida na concentração anômala da ß-catenina no núcleo celular. 1 Já o biomarcador OPN no tecido ósseo normal é vital para desenvolvimento do osteoblasto. E o marcador ABCB5 é um novo transportador humano de membrana do grupo de proteínas ABC, que foi identificado e caracterizado na pele humana.

O presente trabalho objetiva correlacionar a expressão, por imunoistoquímica, das proteínas OPN, ABCB5 e WNT3A de material anatomopatológico, obtido de blocos de parafina e/ou lâminas, em pacientes com OS, analisando características epidemiológicas, sua presença e influência na evolução e progressão da doença.

## Materiais e métodos

O presente trabalho foi aprovado pelo comitê de ética da instituição sob o número CAAE: 69385417.7.1001.0103.

Dados e anátomo-clínicos foram obtidos a partir do prontuário eletrônico do sistema Philips Tasy Electronic Medical Record (Philips Healthcare, Best, Holanda) do hospital, e a revisão dos prontuários físicos e de laudos anátomo-patológicos, pelo serviço de anatomia patológica dos 64 pacientes portadores de OS atendidos no período de 2005 e 2017.

A busca inicial foi por meio da Classificação Internacional de Doenças (CID), código C40, e as anotações clínicas de cada caso, por protocolo padronizado com as variáveis nome, idade do paciente ao diagnóstico, sexo, número de tumores, número do bloco de parafina, diagnóstico histológico, local do tumor primário, data do diagnóstico, presença de metástases, local da metástase ao diagnóstico (se houvesse), tempo de sobrevida em meses, evento óbito (se houvesse), tempo de seguimento, e progressão da doença.

O término da data de avaliação da sobrevida dos pacientes foi dezembro de 2017.

## Progressão da doença e tempo de sobrevida

Alguns pacientes, mesmo na vigência do tratamento, apresentaram recidiva da neoplasia ou progressão da doença com piora do estadiamento clínico, seja por retorno do tumor no sítio cirúrgico, ou aparecimento de metástase local ( *skip* ) ou à distância, em vísceras ou linfonodos. Os locais da progressão à distância registrados foram o pulmão e o linfonodo.

Posteriormente, calculou-se em meses o tempo livre de doença, que consiste no período que o paciente permaneceu sem sinais ou sintomas da neoplasia.

Em relação ao tempo de sobrevida, foi calculado pelo óbito ou data da última avaliação. Subtraiu-se a data em que foi realizado o diagnóstico de OS com a data do óbito ou última avaliação.

## Seleção das amostras

Após a seleção inicial dos casos, ocorreu a busca dos respectivos blocos de parafina, os quais, foram selecionados somente os que possuíssem massa tumoral suficiente para serem realizados cortes adicionais sem que se usasse totalmente o material biológico.

As lâminas dos tumores foram reavaliadas por um segundo patologista independente, com o intuito de confirmar o diagnóstico de OS.

Para os blocos que não possuíssem lâmina de hematoxilina-eosina (HE), foi requisitada a sua produção. Nos casos em que haviam mais de um bloco de parafina, o patologista escolheu o bloco que possuía maior massa neoplásica.

Os blocos devidamente checados foram enviados para realizar a imunomarcação dos biomarcadores: OPN, WNT3A, ABCB5.

## Imunoistoquímica

A técnica de imunoistoquímica realizada no instrumento Ventana BenchMark *ULTRA* (Roche Diagnostics Corporation, Indianápolis, IN, EUA) com processamento 3 em 1 integrado e preparadas na seguinte ordem: desparafinização; re-hidratação; recuperação antigênica com tampões Cell Conditioning 1 (pH alto) e 2 (pH baixo) (Roche Diagnostics Corporation); os anticorpos primários foram incubados por 16 a 20 minutos em temperatura ambiente; em seguida, as lâminas foram submetidas à técnica da imunoperoxidase e foi realizada a amplificação da marcação através do Kit UltraView Universal DAB Detection (Ventana Medical Systems, Oro Valley, AZ, EUA); e, após realizada a imunomarcação, as lâminas de microarranjo de tecidos ( *tissue microarray* – TMA, em inglês) foram laudadas. Controles positivos internos e externos testaram a fidelidade das reações.

## Anticorpos utilizados

Os anticorpos primários utilizados estão descritos na Tabela 1 .

**Tabela 1:** Descrição dos anticorpos primários com seus respectivos fabricantes e diluições

Biomarcador	Anticorpo primário	Fabricante	Diluição
ABCB5	Anti-ABCB5, clone 5H3C6	*GeneTex*	1:200
OPN	Anti-Osteopontin, policlonal	*Anti-Osteopontin, policlonal*	1:25
WNT3A	Anti-WNT3A, policlonal	*GeneTex*	1:400

**Abreviação:** OPN, osteopontina. **Fonte:** Os autores (2019).

## Laudo da imunomarcação

As lâminas foram laudadas no microscópio Olympus CX31 (Olympus Life Science, Waltham, MA, EUA) por 2 patologistas diferentes, em tempos diferentes, utilizando os seguintes parâmetros:

- **Positivo:** Presença combinada do anticorpo ABCB5 na membrana citoplasmática e no citoplasma; presença do anticorpo WNT3A no citoplasma; e presença do anticorpo OPN no citoplasma.
- **Negativas:** Ausência de expressão da marcação cromógena nos alvos dos referidos anticorpos.
- **Inconclusivo:** Impossibilidade de avaliar a expressão dos anticorpos, devido a problemas pré-analíticos (má-fixação, descalcificação ácida e parafina inadequada).

## Resultados

As informações relativas aos pacientes estudados foram apresentadas com: dados de prontuário, número do paciente, lâminas, idade ao diagnóstico, gênero e local do tumor sobre amostra estudada ( Tabela 2 ). Analisamos 40 amostras de blocos de parafina de espécimes de biopsias e peças cirúrgicas de tumores primários e metastáticos, retirados de 28 pacientes de OS provenientes do serviço de anatomia patológica, no período de 2005 a 2017. Relativos à sobrevida, presença ou não, local da metástase e expressão dos marcadores ABCB5, WNT3A e OPN ( Tabela 3 ).

**Tabela 2:** Dados: prontuário, número do paciente, lâminas, idade ao diagnóstico, gênero e local

Prontuário	Número do paciente	Lâminas	Idade ao diagnóstico	Gênero	Local
14001945	4	162818B	20	m	MI
	4	167536B / 2-17	20	m	MI
14002913	5	143741B	12	f	MS
1001989	6	143042B	26	f	MI
	6	149162B / 3-11	26	f	MI
	6	157099B	27	f	MI
13000565	7	130884B	13	f	MI
	7	135117	13	f	MI
	7	140955B / 1-5	14	f	MI
	7	143457B	14	f	AXIAL
12006205	8	140430B	62	m	MS
13005621	10	138466B 1-57	19	m	MI
13002093	11	134093B	20	m	MI
	11	135736B	20	m	MI
7002221	12	073238B / 1-2	16	f	MS
	12	073801B	16	f	MS
7002774	13	074648B	16	m	MI
	13	077531B	16	m	MI
6008700	14	0608869E	61	f	MI
7001977	15	074428B	36	f	MI
11003053	34	116772B / 1-8	20	m	MI
11004802	36	116711B	45	m	MS
13001519	38	135295B / 1-25	13	f	MI
5006762	40	056664B	13	f	MI
6002708	43	0602903B	10	f	MI
6001225	45	0606133B / 1	13	f	MI
6008407	47	070309B / 1-3	27	m	MI
5004056	48	0600157B / 1-2	23	m	MS
15000164	51	150432B / 1-2	21	f	MI
14006899	52	154918B / 2-7	18	m	MI
14007558	53	150955B / 3-20	14	f	MI
15006646	55	167192B / 1-15	26	f	AXIAL
15007598	56	1610793B / 2-6	32	f	MI
16002529	58	163780B	20	m	MS
	58	168657B / 1-14	20	m	MS
16004018	59	166003B / 1-9	22	m	MS
16009072	62	1612817B / 1-2	16	m	MI
	62	170557B / 1-13	17	m	MI
17008495	63	1714419B	11	m	MI

Abreviações: AXIAL, esqueleto axial; MI, membro inferior; MS, membro superior.

**Tabela 3:** Dados: sobrevida, metástase, local, ABCB5, WNT3A e OPN

Prontuário	Sobrevida (meses)	Metástase	Local	ABCB5	WNT3A	OPN
14001945	57	Sim	Pulmão	Inconclusivo	Inconclusivo	Inconclusivo
	52	Sim	Pulmão	Positivo	Negativo	Negativo
14002913	67	Não		Negativo	Negativo	Negativo
1001989	18	Sim	Pulmão	Positivo	Negativo	Negativo
	12	Sim	Pulmão	Negativo	Negativo	Negativo
	2	Sim	Pulmão	Negativo	Negativo	Negativo
13000565	17	Sim	Pulmão	Positivo	Negativo	Negativo
	12	Sim	Pulmão	Positivo	Negativo	Negativo
	5	Sim	Pulmão	Positivo	Positivo	Negativo
	3	Sim	Pulmão	Inconclusivo	Inconclusivo	Inconclusivo
12006205	72	Não		Positivo	Negativo	Negativo
13005621	3	Não		Inconclusivo	Inconclusivo	Inconclusivo
13002093	11	Sim	Pulmão	Positivo	Positivo	Negativo
	12	Sim	Pulmão	Negativo	Negativo	Negativo
7002221	24	Sim	Pulmão	Inconclusivo	Inconclusivo	Inconclusivo
	24	Sim	Pulmão	Positivo	Negativo	Negativo
7002774	29	Sim	Pulmão	Positivo	Positivo	Negativo
	25	Sim	Pulmão	Positivo	Negativo	Negativo
6008700	16	Não		Negativo	Negativo	Negativo
7001977	124	Não		Negativo	Negativo	Negativo
11003053	75	Sim	Pulmão	Positivo	Negativo	Negativo
11004802	68	Não		Positivo	Negativo	Negativo
13001519	8	Sim	Pulmão	Positivo	Negativo	Negativo
5006762	41	Sim	Pulmão	Inconclusivo	Inconclusivo	Inconclusivo
6002708	128	Não		Positivo	Negativo	Negativo
6001225	16	Sim	Linfonodo	Positivo	Negativo	Negativo
6008407	118	Sim	Pulmão	Inconclusivo	Inconclusivo	Inconclusivo
5004056	133	Não		Positivo	Negativo	Negativo
15000164	55	Sim	Pulmão	Negativo	Negativo	Negativo
14006899	3	Não		Negativo	Negativo	Negativo
14007558	34	Sim	Pulmão	Negativo	Negativo	Negativo
15006646	2	Não		Inconclusivo	Inconclusivo	Inconclusivo
15007598	52	Não		Positivo	Negativo	Negativo
16002529	18	Sim	Pulmão	Inconclusivo	Inconclusivo	Inconclusivo
	13	Sim	Pulmão	Positivo	Negativo	Negativo
16004018	36	Não		Inconclusivo	Inconclusivo	Inconclusivo
16009072	12	Não		Positivo	Negativo	Negativo
	11	Não		Negativo	Inconclusivo	Inconclusivo
17008495	24	Não		Negativo	Negativo	Negativo

**Abreviação:** OPN, osteopontina.

A Tabela 4 apresentada a análise realizada com base nos dados de 40 lâminas em 28 pacientes com OS.

**Tabela 4:** Lâminas pré- e pós-quimioterapia

Paciente	Total	Número de lâminas	
		Tecido ósseo livre de tratamento	Tecido ósseo após terapia
1	1	1	
2	1	1	
3	1		1
4	1	1	
5	1	1	
6	1	1	
7	1	1	
8	1		1
9	1	1	
10	1	1	
11	1		1
12	1		1
13	1		1
14	1	1	
15	1		1
16	1		1
17	1		1
18	1		1
19	1	1	
20	2	1	1
21	2	2	
22	2	1	1
23	2	2	
24	2	1	1
25	2	1	1
26	2	2	
27	3	1	2
28	4	1	3
Total de lâminas	40	22	18

Variáveis relativas ao paciente, como idade ao diagnóstico, sexo, número de lâminas ao longo do período de segmento e óbitos estão descritos na Tabela 5 .

**Tabela 5:** Variáveis relativas ao paciente ( *n* = 28)

Variável	N válidos	Classificação	Resultado*
Idade ao diagnóstico (anos)	28		23 ± 13,4 (10–62)
		Até 20	17 (60,7)
		Mais de 20	11 (39,3)
Sexo	28	Feminino	14 (50)
		Masculino	14 (50)
Número de lâminas ao longo do período de seguimento	28	1	19 (67,9)
		2, 3 ou 4	9 (32,1)
Óbito	28	Não	14 (50)
		Sim	14 (50)
Tempo de seguimento (não óbito) (meses)	14		80,0 ± 42,0 (27,5–150)
Tempo de sobrevida (óbitos) (meses)	14		18,2 ± 12,1 (2,7–42,1)
Tempo de seguimento geral (meses)	28		48,9 ± 43,6 (2,7–150)
Metástase	28	Não	14 (50)
		Sim	14 (50)
Local da metástase (por paciente)	14	Pulmão	13 (92,9)
		Linfonodo	1 (7,1)

*Descrito por média ± desvio padrão (mínimo–máximo) ou por frequência (percentual).

Na Tabela 6 são apresentados os percentuais de sobrevida em cada tempo estimados por Kaplan-Meier.

**Tabela 6:** Taxa de sobrevida em até 5 anos

Tempo (meses)	% de sobrevida
0 (diagnóstico)	100%
3 meses	100%
6 meses	96,4%
1 ano	92,9%
1,5 ano	67,9%
2 anos	64,3%
3 anos	56,9%
4 anos	52,5%
5 anos	46,7%

## Dados descritivos dos marcadores ABCB5, WNT3A e OPN

Estão apresentados resultados (n válidos) relativos a variáveis de localização do tumor de 28 pacientes em membros inferiores (MIs), membros superiores (MSs), esqueleto axial (AXIAL); tipo de lâmina (biopsia, pós-quimioterapia e peça sem quimioterapia) e tipo de tecido da lâmina (sem tratamento e após terapia). Considerou-se apenas uma lâmina por paciente, sendo encontrados os resultados seguintes (n = 28): localização do tumor da lâmina – MIs (n [%] 20 [71,4]), MSs (n [%] 7 [25,0]), AXIAL (n [%] 1 [3,6]); tipo de lâmina – biópsia (n [%] 16 [57,1]), pós-quimio (n [%] 10 [35,7]), sem quimio (n [%] 2 [7,1]); tipo de tecido da lâmina – tecido ósseo livre de tratamento (n [%] 18 [64,3]), tecido ósseo após terapia (n [%] 10 [35,7]).

Os dados descritivos dos marcadores ABCB5, WNT3A e OPN, considerando apenas uma lâmina por paciente e percentuais, apresenta resultados com ABCB5 e WNT3A, após retirados os resultados inconclusivos, conforme segue:

## ABCB5, n válido = 28

Negativo (n [%] 7 [25,0])

Positivo (n [%] 14 [50,0])

Inconclusivo (n [%] 7 [25,0])

## ABCB5, n válido = 21

Negativo (n [%] 7 [33,3])

Positivo (n [%] 14 [66,7])

## WNT3A, n válido = 28

Negativo (n [%] 19 [67,9])

Positivo (n [%] 2 [7,1])

Inconclusivo (n [%] 7 [25,0])

## WNT3A, n válido = 21

Negativo (n [%] 19 [90,5])

Positivo (n [%] 2 [9,5])

## OPN, n válido = 28

Negativo (n [%] 21 [75,0])

Inconclusivo (n [%)] 7 [25,0])

## Avaliação de fatores associados aos resultados dos marcadores e entre fatores e sobrevida

Para esta análise, a unidade de observação foi a lâmina. Lâminas de um mesmo paciente foram consideradas como unidades independentes.

Para cada uma das variáveis, tipo de tecido e localização do tumor, e para cada marcador analisado, testou-se a hipótese nula de que não há associação entre a variável e o marcador versus a hipótese alternativa de que há associação.

Para idade ao diagnóstico e cada marcador, testou-se a hipótese nula de que as médias de idade são iguais para todas as classificações do marcador, versus a hipótese alternativa de que as médias não são iguais.

Com relação à sobrevida, para cada uma das variáveis analisadas, testou-se a hipótese nula de que não há associação entre a variável e a sobrevida, versus a hipótese alternativa de que há associação. São percentuais de óbitos de acordo com as classificações da variável: idade ao diagnóstico, sexo, metástases, local do tumor, marcadores ABCB5, WNT3A, OPN e os valores de *p* dos testes estatísticos.

## Discussão

Os pacientes selecionados portadores de OS foram classificados em Enneking grau IIB ou III.

Na análise do perfil epidemiológico, 17 pacientes apresentavam idade abaixo de 20 anos (pacientes jovens) e 11 acima de 20 anos, sendo que o mais novo tinha apenas 10 anos de idade, em concordância com a literatura. 2 3 4 5 Nesta amostragem, o sexo masculino e o feminino apresentaram a mesma incidência, 50%. Estudos com amostra maiores, demonstraram um discreto predomínio no sexo masculino. 6

Nos pacientes avaliados, quanto à localização das metástases: 14 apresentaram metástases, sendo 13 em tecido pulmonar (92,9%) e 1 nos linfonodos (7,1%). 2 Nesta amostragem, 50% evoluíram com metástases pulmonar. Segundo Evola et al., 7 aproximadamente 20% dos pacientes apresentam metástases pulmonares no diagnóstico inicial, em 40% dos pacientes as metástases ocorrem em um estágio posterior, e 80% das metástases ocorrem no pulmão.

A sobrevida, para esta amostra, foi de 46,7% em 5 anos (60 meses), um pouco abaixo do encontrado na literatura, que gira em torno de 50 a 65%. 2 3 8 Óbito ocorre entre o primeiro e terceiros anos após feito o diagnóstico na maioria dos pacientes, indicando que se trata de uma patologia muito agressiva. 5

A localização em MIs ocorreu em 20 pacientes (71,4 %), MSs em 7 pacientes (25%), 1 caso AXIAL (3,6%). Plenamente em concordância com a literatura. 2 5 7 9 10

As variáveis epidemiológicas (idade ao diagnóstico, sexo, metástases, local e marcadores) não apresentaram significância estatística quando se analisa desfecho óbito. A variável metástase demostra uma tendência à significância ( *p* = 0,086); porém, a amostra é pequena para esta conclusão.

O marcador ABCB5 teve expressão positiva em 14 pacientes (66,7) em 7 pacientes negativas (33,3%), quando se excluíram os inconclusivos que foi em número de 7. Já o WNT3A teve expressão positiva em 2 pacientes (9,5%), em 19 com expressão negativas (90,5%), quando se excluíram os inconclusivos. E o marcador OPN só apresentou lâminas negativas, não houve expressão, tanto em lâminas de tecidos que receberam ou não, quimioterapia neoadjuvante.

Quando se avaliou a expressão de marcadores ABCB5 e WNT3A versus localização do OS em MSs e MIs, faixa etária abaixo ou acima de 20 anos, expressão positiva ou negativa também não apresentou significância. Porém, o marcador OPN só apresentou lâminas negativas tanto em lâminas do membro superior como do membro inferior, e nas faixas etárias acima e abaixo de 20 anos.

Na avaliação das lâminas dos 28 pacientes elegíveis, 6 pacientes tiveram as lâminas examinadas pré e pós-tratamento quimioterápico. Na avaliação pré-tratamento, havendo inicialmente uma lâmina por paciente e as avaliações pós-tratamento quimioterápico, foram analisadas lâminas de acordo com os eventos de progressão da doença (recidivas e metástases), que resultaram em análises de novas lâminas referentes a estes eventos. Portanto, o número de lâminas, pós-tratamento foi superior ao número de lâminas pré-tratamento – 9 lâminas.

Para o marcador ABCB5 na avaliação pré-tratamento, 3 lâminas (50%) apresentaram-se positivas, nenhuma negativa (0%) e 3 inconclusivas (50%). Após o tratamento quimioterápico, 6 apresentaram-se positivas (66,7%), 2 negativas (22,2%) e somente 1 inconclusiva (11,1%). O ABCB5 é um marcador que avalia a resistência às drogas quimioterápicas, e é mediador de quimiorresistência, identificado e comprovado como importante e específico à droga doxorrubicina. Na presente amostra, os pacientes que participaram de esquemas quimioterápicos, também receberam a doxorrubicina; logo, a expressão desta proteína se fez presente nas lâminas pré-tratamento, com 50%, aumentando sua expressão em lâminas pós-tratamento, com 66,7%. Mantendo a hipótese de que células-tronco tumorais desenvolvem a capacidade de resistência a drogas citotóxicas, não se conseguiu inibir a progressão tumoral. 11 12 13

Haydon et al. 14 concluíram que a desregulamentação da via WNT/ ß-catenina é uma ocorrência muito comum no OS. Porém, nesta amostra, somente dois pacientes apresentaram indícios de ativação desta via. Mas, quando isso ocorreu, foi justamente com pacientes de pior prognóstico. Não houve significância estatística, quando se considerou a expressão dessa proteína influenciando na sobrevida.

O OPN não foi detectado nos osteoblastomas e nas peças de remodelação óssea. Sua expressão não teve influência nos pacientes em geral ou na sobrevida livre de doença. E não fornece informações preditivas sobre o resultado de pacientes com OS. A formação e a diferenciação do osteoblasto são fundamentais para formação do tecido ósseo. Além disso, o OPN interfere na diferenciação da célula primária mesenquimal em pré-osteoblasto, gerando osteoblasto maduro. A interrupção deste processo pode ser a causa principal da formação do OS. Portanto, Luo et al., 15 relatam diminuição dos níveis de OPN em OS, pois não atua na diferenciação dos osteoblastos. Expressão da proteína OPN foi negativa em 100% das lâminas, corroborando os achados desses autores.

## Conclusão

Os biomarcadores ABCB5, OPN, WNT3A, em estudo imunoistoquímico no OS, apresentaram-se ausentes de significância estatística nos parâmetros epidemiológicos. Não demonstraram ser um fator preditor ou de agressividade para o OS. Observou-se discreto aumento de ABCB5, pós-quimioterapia. O bloqueio da expressão, desta proteína ABCB5 suporta a hipótese de aumento de sobrevida, livre de recidiva da doença. A proteína OPN não se mostrou presente na etiologia e no desenvolvimento do OS. Neste estudo, a via canônica WNT/ß-catenina não fez presente de maneira convincente pela expressão do marcador WNT3A. Estudos prospectivos, com material de banco de tumores congelados, randomizados e controlados, são pertinentes para consolidar e promover avanços em futuras pesquisas.
